# *Mycobacterium avium* subsp. *hominissuis* Infection in Swine Associated with Peat Used for Bedding

**DOI:** 10.1155/2014/189649

**Published:** 2014-09-15

**Authors:** Tone Bjordal Johansen, Angelika Agdestein, Bjørn Lium, Anne Jørgensen, Berit Djønne

**Affiliations:** ^1^Norwegian Veterinary Institute, P.O. Box 750, Sentrum, 0106 Oslo, Norway; ^2^Animalia, P.O. Box 396, Økern, 0513 Oslo, Norway

## Abstract

*Mycobacterium avium* subsp. *hominissuis* is an environmental bacterium causing opportunistic infections in swine, resulting in economic losses. Additionally, the zoonotic aspect of such infections is of concern. In the southeastern region of Norway in 2009 and 2010, an increase in condemnation of pig carcasses with tuberculous lesions was seen at the meat inspection. The use of peat as bedding in the herds was suspected to be a common factor, and a project examining pigs and environmental samples from the herds was initiated. Lesions detected at meat inspection in pigs originating from 15 herds were sampled. Environmental samples including peat from six of the herds and from three peat production facilities were additionally collected. Samples were analysed by culture and isolates genotyped by MLVA analysis. *Mycobacterium avium* subsp. *hominissuis* was detected in 35 out of 46 pigs, in 16 out of 20 samples of peat, and in one sample of sawdust. MLVA analysis demonstrated identical isolates from peat and pigs within the same farms. Polyclonal infection was demonstrated by analysis of multiple isolates from the same pig. To conclude, the increase in condemnation of porcine carcasses at slaughter due to mycobacteriosis seemed to be related to untreated peat used as bedding.

## 1. Background

*Mycobacterium avium* subsp.* hominissuis*, a member of the* M. avium* complex, is regarded as an opportunistic pathogen for pigs and humans [1]. Infection in pigs is typically characterised by granulomatous lesions in lymph nodes associated with the digestive system, but lesions in internal organs like the liver, lungs, and kidneys may also occur. The lesions are usually discovered at meat inspection and can imply serious economic losses for the producer if detected in several pigs and in multiple organs [2, 3]. Occasionally, clinical symptoms like wasting and abortion are seen [4]. The gross pathological presentation of lesions is not possible to distinguish from those caused by* M. bovis*, causing issues of proper management of carcasses and the herds of origin before the diagnosis is confirmed. In humans,* M. avium* subsp.* hominissuis* is a known cause of systematic infections in immunocompromised patients, lung infections in patients with underlying pulmonary disorders, and lymphadenitis in the head and neck region of children. A zoonotic aspect of* M. avium* infections has not been ruled out [3].

Nontuberculous mycobacteria, like* M. avium* subsp.* hominissuis*, are known to be ubiquitous in the environment, where they are able to survive and multiply [5, 6]. They have been isolated from a variety of environmental samples, like water, food, soil, sawdust, and peat [7–15]. In the Norwegian pig production, sawdust, wood shavings, and peat are materials commonly used for bedding. Peat has become more popular as bedding material, due to the higher costs and limited accessibility of sawdust and wood shavings. Additionally, peat is used as a feed supplement for piglets, both as iron enrichment for suckling piglets and for regulation of intestinal function in newly weaned piglets [16]. Contaminated peat and sawdust have been associated with outbreaks of* M. avium* subsp.* hominissuis* infections in swine as confirmed by molecular fingerprinting methods [7, 9, 11, 14, 15].

In the southeastern region of Norway, starting in December 2009 and lasting through the beginning of the year 2010, there was an increase in the number of condemnations of swine carcasses due to tuberculous lesions in lymph nodes, liver, and lungs. Several herds were involved and some had involvement of multiple carcasses. A common factor for many of the herds was the use of peat as bedding material. It was, therefore, hypothesised that peat might be the cause of mycobacterial infection in these herds, and a project examining lesions detected at meat inspection as well as environmental samples, including peat, from the herds and from peat production facilities was initiated.

## 2. Materials and Methods

The majority of pigs included in the study were slaughtered at Furuseth AS located in the county Akershus. Additionally, some of the pigs were slaughtered at Nortura Sarpsborg in Østfold. The animals originated from seven counties in the southeastern part of Norway: Østfold, Vestfold, Buskerud, Telemark, Akershus, Hedmark, and Oppland. This was a descriptive study conducted in order to clarify the infection status of the herds, and sampling was, therefore, not randomized and systematically performed, but based on inclusion of the samples sent to the laboratory. Forty-six pigs with gross lesions indicating mycobacterial infection and originating from 15 herds (A–I and K–P) were sampled, and tissue samples were sent to the Norwegian Veterinary Institute for analysis. Only carcasses showing visible lesions at regular meat inspection were sampled. From each pig, lymph nodes, liver, and/or lungs were sampled. Twenty-three environmental samples, including peat intended for bedding, sawdust, hay/straw, and water, were collected from six of the herds (A, B, I, J, O, and P). Additionally, 16 samples of peat intended for bedding were retrieved from three different production facilities (facilities I, II, and III), of which peat producer II delivered peat to farm B and producer III to farm A. Two samples drawn from peat intended as feed supplement for piglets were also examined (facility IV) (Table 1).

Isolation of mycobacteria from organ and environmental samples was performed as described earlier on slants of Middlebrook 7H10 (BD Diagnostics, Sparks, MD) w/10% oleic acid (BD Diagnostics) with and without antibiotics and fungicides (final concentrations of 100 *μ*g/mL carbenicillin, 200 U/mL polymyxin B sulphate, 19.5 *μ*g/mL trimethoprim lactate, and 10 *μ*g/mL amphotericin B), Dubos P, and Stonebrink's medium [7, 15]. Slants were incubated for eight weeks at 37°C, and colonies resembling mycobacteria were subcultured on Middlebrook 7H10 and the medium they were initially observed to grow on. On primary isolation, attempt was made to pick one colony of each morphotype, when more than one was present. When more than one organ/lymph node from the same pig was positive, one isolate from each organ was included for further analysis. Also from some environmental samples, more than one isolate was examined further. Isolates shown to be acid-fast rods by the Ziehl-Neelsen (ZN) staining method were identified as* M. avium* by Accu Probe (GenProbe Inc., San Diego, CA), and further determination of subspecies was based on the presence or absence of IS*901* and IS*1245*, analysed by PCR using 1U AmpliTaq DNA polymerase (Applied Biosystems, Foster City, CA). Primers 901a and 901c were used for the amplification of IS*901* and primers P40 and P41 for IS*1245* [18, 19]. PCR conditions were set as described earlier [20]. The reference strain* M. avium* subsp.* avium* ATCC 25291 was included as a positive control and MQ water as negative control. Acid fast bacteria not identified as* M. avium* were analysed by 16S rDNA sequencing as described previously [21].

Isolates identified as* M. avium* subsp.* hominissuis* were analysed by multiple locus variable number of tandem repeat analysis (MLVA), also referred to as MIRU-VNTR typing, using the eight loci as described by Thibault et al. [17]. Product size of PCR fragments was analysed by capillary electrophoresis using the Agilent Bioanalyzer (Agilent Technologies, Santa Clara, CA, USA) as described [7].* Mycobacterium avium* subsp.* avium* ATTC 25291 was used as a control in each run. Sizes of the PCR products were converted to a corresponding tandem repeat number for each locus as described by Thibault et al. [17]. The data was entered into BioNumerics version 6.1 (Applied Maths, Sint-Martens-Latem, Belgium), and cluster analysis was performed using the categorical method and the unrooted UPMGA tree. Only isolates of 100% similarity, that is, isolates having the same number of tandem repeats in each locus, were assigned to the same cluster. The HG index/diversity index was calculated as described [22, 23], using the Discriminatory Power Calculator (http://insilico.ehu.es/mini_tools/discriminatory_power/index.php).

When more than one organ from the same pig was positive for* M. avium* subsp.* hominissuis*, the MLVA profile of the isolates was subjected to minimum spanning tree (MST) analysis in BioNumerics 6.1, illustrating the relationship and possible mutation pathways within the clusters based on single locus variations (SLV).* Mycobacterium avium* subsp.* avium* ATCC 25291 was used as a reference strain. MST is a tool that takes a unidirectional graph and extracts the subgraphs with the smallest weights [24, 25]. The MST was created based on the MLVA data used for the cluster analysis of the complete dataset. The nodes (circles) consist of identical genotypes and the edges (lines) of weights based on number of mutations (steps) taken from the loci used. Long weights (steps) indicate multiple mutations, while short weights indicate few mutations. The MST algorithm was then applied to this graph to extract all subgraphs with the minimal overall weight sum. Hence, the most similar strains are clustered closely together with short and thick edges, while increasing genomic variation leads to thin and longer edges.

## 3. Results

Thirty-five out of the 46 slaughtered pigs sent for analysis showed positive growth of mycobacteria. These 35 pigs originated from 12 herds. All together 72 isolates were obtained from different organs from the 35 pigs. All isolates from pigs were verified as* M. avium* subsp.* hominissuis* based on identification with Accu Probe, absence of IS*901* and presence of IS*1245*.* Mycobacterium avium* subsp.* hominissuis* was also detected from 16 of 20 samples of peat intended for bedding (herds A, B, I, and J and peat producers I, II, and III) and in one out of four samples of sawdust (herd A) (Table 1). From three of the herds* M. avium* subsp.* hominissuis* was not detected, neither from peat (herds O and P) nor from pigs (herds N, O, and P). None of the samples of hay/straw or water were positive for mycobacteria. The two samples of peat intended as feed supplements for piglets were also negative for mycobacteria. Additionally, seven samples of peat intended for bedding were positive for* M. bohemicum*, one sample of peat showed growth of* M. palustre,* and two peat samples showed growth of* Mycobacterium* sp. that could not be further identified with the methods used in the current study.

**Figure 1:**
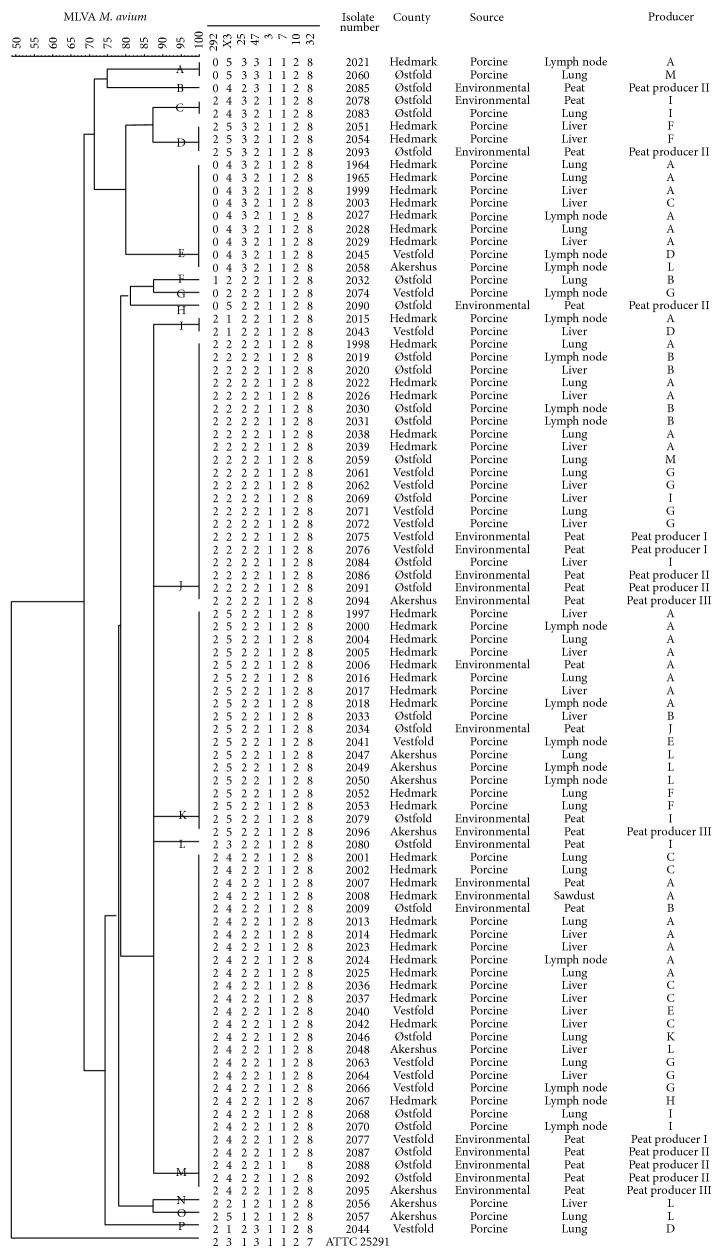
An unrooted tree showing genetic relationship between isolates of* Mycobacterium avium* subsp.* hominissuis* originating from peat, sawdust, and lymph nodes from slaughtered pigs in Norwegian herds. The dendrogram is based on eight-locus MLVA analysis [17]. The tree was created in BioNumerics 6.1, using categorical data and the unweighted pair group method with arithmetic mean (UPGMA).* Mycobacterium avium* subsp.* avium* ATCC 25291 was used as a reference strain. The different MLVA profiles are named A–P.

All isolates from pigs (*n* = 71), peat (*n* = 22), and sawdust (*n* = 1) underwent MLVA analysis. Two isolates, one from peat and one porcine isolate, were excluded from analysis due to double amplification product in one locus. MLVA analysis identified 16 different profiles among the 92 analysed isolates, distributed on eight clusters and eight singletons (Figure 1). Clusters were recognised when containing ≥2 isolates with identical profile. All tandem repeats were present in the isolates analysed, except for TR10 which was lacking in one isolate. The range and mode for the different tandem repeats were as follows: TR292 (range 0–2, mode 2), TRX3 (1–5, 4), TR25 (2-3, 2), TR47 (2-3, 2), TR3 (1-1, 1), TR7 (1-1, 1), TR10 (2-2, 2), and TR32 (8-8, 8). The discriminatory index for MLVA was calculated to 0,819.

Four of the VNTR loci were monomorphic markers (TR3, TR7, TR10, and TR32). Of these, TR7 had an amplicon size of between 180 and 200 bp, which is between one and two copies as described by Thibault et al. [17], but as it has been experienced that the size of these amplicons differs between* M. avium* subsp.* hominissuis* and* M. avium* subsp.* paratuberculosis*, the experienced amplicon size corresponds to one copy of TR7 as described [26, 27].

For illustration, each MLVA profile was labelled from A to P (Figure 1). Not only identical porcine isolates from the same farm but also identical isolates from pigs from different farms were detected. On several occasions, environmental isolates and porcine isolates were found to be identical. From farm A, one isolate from peat (number 2007) and one from sawdust (number 2008) were identical to five porcine isolates originating from three pigs (numbers 2013, 2014, 2023, 2024, and 2025) (MLVA profile M). Additionally, another isolate from peat from the same farm (number 2006) was identical to seven porcine isolates from four pigs (numbers 1997, 2000, 2004, 2005, 2017, 2016, and 2018) (profile K). This farm received peat from peat producer III, where isolates with the same two profiles were detected (number 2095 with profile M and number 2096 with profile K). Farm B used peat from peat producer II, and identical isolates from pigs and from this peat producer were detected. Four porcine isolates from two pigs (numbers 2019, 2020, 2030, and 2031) showed identical MLVA profiles with isolates from peat producer II (number 2086 and number 2091) (profile J).

**Figure 2:**
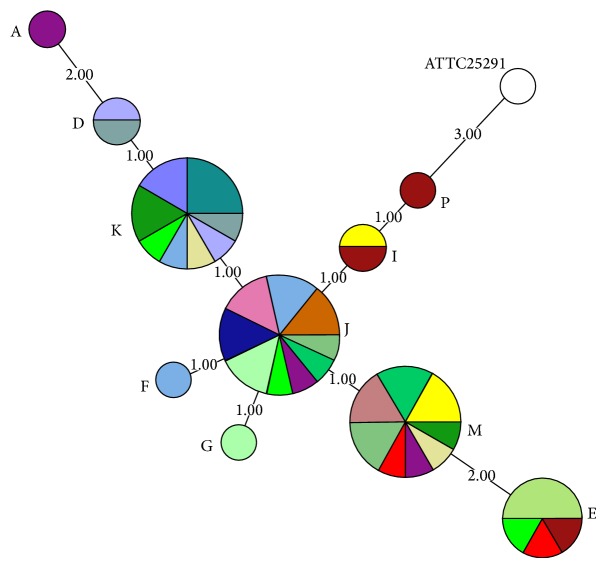
Minimum spanning tree (MST) analysis made in BioNumerics 6.1 illustrating the relatedness of isolates when more than one organ from the same pig was positive on culture for* Mycobacterium avium* subsp.* hominissuis*. All isolates (*N* = 52) originating from 20 pigs were subjected for analysis. The figure illustrates the relationship and possible mutation pathways within the clusters based on single locus variations (SLV). The MST was created based on the MLVA data used for the cluster analysis of the complete dataset. The nodes (circles) consist of identical genotypes and the edges (lines) of weights based on number of mutations (steps) taken from the loci used. Long weights (steps) indicate multiple mutations, while short weights indicate few mutations. Isolates originating from the same pig are illustrated in the same color. The size of the nodes represents the number of isolates showing the same genotype, and the size of the colored fields represents the number of isolates from the same pig within the nodes. Each node is labelled with the letter describing the MLVA profile as shown in Figure 1.* Mycobacterium avium* subsp.* avium* ATCC 25291 was used as a reference strain.

Isolates originating from different organs from the same pig did on several occasions show differences in MLVA profiles. In all, 52 isolates from 20 pigs were compared by MST (Figure 2). Some isolates originating from the same pig showed difference in only one locus, exemplified by isolate number 2022 (profile J) and number 2023 (profile M), where there was a difference in the number of repeats in locus X3 (Figure 1). Other isolates from one pig, like number 2021 (profile A) and number 2022 (profile J), as well as number 2044 (profile P) and number 2045 (profile E), differed in multiple loci (Figure 2).

## 4. Discussion

Peat is used both as bedding material for piglets, grower pigs, and finisher pigs and as feed additive providing iron supplement and intestinal regulation for piglets. However, even when used as bedding material, pigs will often ingest some of the peat. If the peat is contaminated with mycobacteria, risk of infection is increased, especially if ingested by young animals. The presence of* M. avium* subsp.* hominissuis* in the majority of samples of peat intended for bedding, together with the detection of identical isolates from swine and peat in some of the herds, confirmed that peat is a product capable of introducing the infectious agent into the pig herds. Such massive infection pressure might cause condemnation of carcasses as slaughter, which is an economic concern for the farmer. It has additionally been proven that pig herds with* M. avium* infections can have unapparent animals at slaughter that still harbour* M. avium* subsp.* hominissuis* in lymph nodes [7, 15]. As long as the zoonotic aspect of* M. avium* infections is not ruled out, this might be of concern for the pig industry.

This study has certain limitations, being mainly descriptive and, therefore, lacking randomized and systematic sampling. Environmental samples were only collected from six herds, while pigs were sampled from fifteen herds. Additionally, information about management was not obtained from all herds. It is, therefore, difficult to draw firm conclusions about the source of* M. avium* subsp.* hominissuis* for all the herds in the study. In two of the involved farms, however, isolates from peat sampled at the factory supplying the farm, from the peat intended for bedding, and from slaughtered pigs were of the same genotype, which is yet another indication of peat being the probable source of infection for these farms. The results are in concordance with other publications that document the presence of* M. avium* subsp.* hominissuis* in peat [7, 9, 15].

Other species of mycobacteria were also detected in peat samples. Both* M. bohemicum* and* M. palustre* have previously been detected in both peat and lymph nodes from swine and can cause lesions similar to those caused by* M. avium* [7, 28]. No mycobacteria were detected in the peat intended as a feed supplement. Such peat is treated with acetic acid and formic acid to control the microbial flora but not heat treated. Mycobacteria would probably survive such treatment. The production site for this peat was different from the factories producing peat for bedding included in the study. However, as only two samples of this type of peat were analysed, no firm conclusions can be made regarding the risk factor of this feed additive when it comes to mycobacteriosis in pigs.

Peat seems to be a habitat where mycobacteria, including* M. avium,* thrive. Low pH, low oxygen content, and high organic matter are factors that have been correlated with increased levels of mycobacteria in soil samples, suggesting that peat might provide excellent conditions for* M. avium* [5, 29, 30]. Peat has many positive qualities in the pig production like the ability to bind ammonium, water, and urine, thereby improving the animals' environment and reducing the risk of diseases like joint infections and diarrhoea [31]. The cost for the farmer is also low. On the downside is the risk of infectious agents that may be introduced by peat like mycobacteria and pathogenic fungi [16, 31], which makes increased knowledge about the frequency of mycobacteria in peat essential for an adequate risk-benefit analysis of the use of peat in the pig production. Also, the age of the pigs at the time of peat introduction might be of importance, as young animals have a weak immune system and are more at risk of infections.

One sample of sawdust showed growth of* M. avium* subsp.* hominissuis*, which is in concordance with findings from other studies [11, 15]. The other environmental samples analysed were negative for mycobacteria, although one could assume that a higher sample volume would allow detection of mycobacteria in such samples. Water, in particular, has previously been described as a source of* M. avium* subsp.* hominissuis* for both humans and pigs [6, 14]. However, the detection frequency of mycobacteria in the other environmental samples, when compared to peat, suggests that these types of bedding materials might be a safer choice for the farmer.

The study demonstrated a large proportion of pigs infected with* M. avium* subsp.* hominissuis*, and in multiple cases isolates with different MLVA profile were detected from the same animal. Such findings have been described by other authors analysing isolates from both pigs and humans [27, 32]. Also for other mycobacteria, as* M. avium* subsp.* paratuberculosis* and* M. tuberculosis*, the same phenomenon has been described [24, 25, 33]. The finding of genetic different isolates based on MLVA from the same animal could be a result of mutation of the strain during the course of infection or of coinfection with multiple isolates. When the MLVA profiles of the isolates differ only by one locus, mutation during infection could explain the observed difference. However, when isolates differ on more than one locus, polyclonal infection is a more likely explanation, as the alternative would have to be multiple mutations occurring in the same strain during infection. These findings could indicate a large infection pressure in the herd, probably caused by contaminated peat.

The eight-locus MLVA method used in this study is a rapid PCR based typing method well suited for discrimination of bacterial isolates. The discriminatory power experienced in the present study is slightly reduced compared to what has been described by others [27, 32, 34]. This could be explained by the epidemiologic link between the isolates, as multiple isolates were retrieved from the same farms and production sites and also from the same pigs. Not all loci are equally suited for discrimination. Four monomorphic markers were described in this study (TR3, TR7, TR10, and TR32). Of these, three have showed a low allelic diversity for isolates of* M. avium* subsp.* hominissuis* in other studies, while TR3 has been demonstrated as monomorphic also in other studies [17, 26, 32, 35]. The employment of these markers in this MLVA analysis is, therefore, not adding as much information as the more diverse loci, and the tandem repeats could be excluded or replaced with other targets, such as one or more of the tandem repeats used in the MATR-VNTR described by Inagaki et al. [26].

To conclude, the increase of condemnation of porcine carcasses at slaughter due to* M. avium* subsp.* hominissuis* experienced by the Norwegian pig industry in 2009 to 2010 seemed to be related to contaminated peat used as bedding in the herds. As a result of the findings, the use of peat was reduced in most herds and the situation stabilized. Pig farmers that consider use of peat in their herds must be aware of the risk for mycobacteriosis.

## Figures and Tables

**Table 1:** Samples examined for mycobacteria.

Sampled material	Number examined	Number positive for *M. avium* subsp. *hominissuis *
Pigs (organ samples)	46 (91)	35 (72)
Peat intended for bedding^a^	4	4
Peat intended for bedding^b^	16	12
Peat intended for feed supplement^b^	2	0
Sawdust^a^	4	1
Hay/straw^a^	5	0
Water^a^	10	0

^a^Sampled at farms.

^b^Sampled at peat production facilities.
